# Urban people’s preferences for life-sustaining treatment or artificial nutrition and hydration in advance decisions

**DOI:** 10.1186/s12910-024-01060-w

**Published:** 2024-05-18

**Authors:** Yi-Ling Wu, Tsai-Wen Lin, Chun-Yi Yang, Samuel Shih-Chih Wang, Sheng-Jean Huang

**Affiliations:** 1 Master’s Program of Transition and Leisure Education for Individuals With Disabilities, University of Taipei, Taipei, Taiwan; 2 National Academy Educational Research, Taipei, Taiwan; 3 Department of Social Work, Taipei City Hospital, Taipei, Taiwan; 4 Department of Health and Welfare, University of Taipei, Taipei, Taiwan; 5 Department of Surgery, Medical College, National Taiwan University Hospital, Taipei, Taiwan

**Keywords:** Advance care planning, Advance decision, Artificial nutrition and hydration, Life-sustaining treatment

## Abstract

**Background:**

The Patient Right to Autonomy Act (PRAA), implemented in Taiwan in 2019, enables the creation of advance decisions (AD) through advance care planning (ACP). This legal framework allows for the withholding and withdrawal of life-sustaining treatment (LST) or artificial nutrition and hydration (ANH) in situations like irreversible coma, vegetative state, severe dementia, or unbearable pain. This study aims to investigate preferences for LST or ANH across various clinical conditions, variations in participant preferences, and factors influencing these preferences among urban residents.

**Methods:**

Employing a survey of legally structured AD documents and convenience sampling for data collection, individuals were enlisted from Taipei City Hospital, serving as the primary trial and demonstration facility for ACP in Taiwan since the commencement of the PRAA in its inaugural year. The study examined ADs and ACP consultation records, documenting gender, age, welfare entitlement, disease conditions, family caregiving experience, location of ACP consultation, participation of second-degree relatives, and the intention to participate in ACP.

**Results:**

Data from 2337 participants were extracted from electronic records. There was high consistency in the willingness to refuse LST and ANH, with significant differences noted between terminal diseases and extremely severe dementia. Additionally, ANH was widely accepted as a time-limited treatment, and there was a prevalent trend of authorizing a health care agent (HCA) to make decisions on behalf of participants. Gender differences were observed, with females more inclined to decline LST and ANH, while males tended towards accepting full or time-limited treatment. Age also played a role, with younger participants more open to treatment and authorizing HCA, and older participants more prone to refusal.

**Conclusion:**

Diverse preferences in LST and ANH were shaped by the public’s current understanding of different clinical states, gender, age, and cultural factors. Our study reveals nuanced end-of-life preferences, evolving ADs, and socio-demographic influences. Further research could explore evolving preferences over time and healthcare professionals’ perspectives on LST and ANH decisions for neurological patients..

## Background

Advance care planning (ACP) is a multifaceted issue involving ethical considerations, humanity, technology, social values, patient preferences, family requests, legislative rationality, and the roles and the obligations of medical professionals [1–5]. Historically, withdrawing the life-sustaining treatment (LST) and artificial nutrition and hydration (ANH) were widely considered a form of murder in many countries [4]. This is primarily due to the contention that the two prerequisites for withdrawal—physician duty of care and patient autonomy—were not met [4]. Decisions to forego LST and ANH are typically made when the patients have little hope of recovery or are approaching the end of life (EoL) [2]. While choosing not to receive treatment is now considered a valid option, it is crucial for patients to express their wishes. Family decisions are assumed to align with the patient’s preferences only if they carefully consider the patient’s perspective [2, 6].

Taiwan has played a leading role in raising the public awareness and enacting legislation on palliative care and ACP in Asia [4]. Over the past two decades, Taiwan, Japan, and Korea have established regulations for withholding and withdrawing LST and ANH [2, 4]. Taiwan, ahead of many Asian countries, has enacted two significant laws related to EoL care. Developed countries like the United Kingdom, the United States, and English-speaking countries such as Australia and Canada have long implemented public regulations on refusing of LST and ANH for EoL care.

The introduction of the Hospice Palliative Care Act (HPCA) in Taiwan in 2000 allowed terminally ill individuals to refuse LST treatment. Subsequent legal amendments permitted the withdrawal of LST upon completion of a medical consent form by the patient or a family representative. Despite progress, regulations in Taiwan did not fully recognize patient autonomy. Physicians were not obligated to communicate the truthfully, disclose diagnoses, or seek agreement from patients [7]. Consequently, the decision to withhold or withdraw LST often fell to family members rather than patients themselves [6, 8]. The HPCA primarily protected the rights of terminal patients, excluding non-terminal patients in vegetative or long-term coma relying on respirators for life-sustaining, who lacked the option to refuse or reject LST under this regulation [9]. In response to these shortcomings, the Patient Right to Autonomy Act (PRAA) was passed in December 2015.

As the first patient-centric legislation in Asia and Taiwan, the PRAA came into effect on January 6, 2019. It empowers individuals with a full capacity to sign advance decisions (ADs) through the advance care planning (ACP) procedure. In alignment with the patients’ right to information, the PRAA explicitly mandates the medical facilities and physicians to inform patients about their medical circumstances, treatments, procedures, prescriptions, and prognoses.

The PRAA permits the discontinuation of LST and ANH for patients with a valid AD who meet one of five clinical conditions: terminal illness, irreversible coma, persistent vegetative state, severe dementia, or other medical disorders specified by the Ministry of Health and Welfare’s (MHW) ordinance. These specified medical disorders encompass unbearable sufferings, incurable diseases, and the absence of alternative treatment options. The AD serves to safeguard the patient’s right to medical autonomy, allowing them to refuse medical treatment in more specific clinical circumstances, including those outlined in the MHW.

In Taiwan, an AD is a formal document wherein individuals, following ACP consultations, express their willingness to accept or reject LST and ANH under specific clinical conditions. Currently, the MHW has officially approved these conditions for five specific clinical scenarios. In accordance with the PRAA, the MHW has designated specific healthcare institutions to provide ACP services. Individuals can attend these authorized institutions to engage in ACP, following which they are eligible to execute the AD.

The ACP process is facilitated by counseling teams composed of physicians, nurses, social workers, or counseling psychologists in authorized institutions. These teams engage in discussions covering medical, social, family, and psychological aspects. Individuals are under no obligation to sign the AD after counseling. Counselors must complete the officially designated training program, which includes an understanding of the conceptual framework and mechanism design related to the PRAA regulations. Additionally, counselors develop proficiency in the skills essential for the ACP counseling process and engage in discussions on clinical practice cases and common issues.

An ACP procedure is a legal requirement for the validity of an AD. LST includes crucial medical interventions capable of prolonging a patient’s life, such as cardiopulmonary resuscitation, mechanical life support systems, blood products, specialized treatments for specific diseases, and antibiotics administered during severe infections. Concrete examples encompass actions like chest compressions, intubation, defibrillation, respiratory support through ventilators, hemodialysis machines, liver support devices, blood transfusions, and antibiotic therapies.

Among the EoL wishes expressed by healthy individuals in the UK, the most widely discussed and essential topic is dying with dignity [5]. EoL-related communications and ACP adoption in East Asian nations are relatively low. The acceptance of ACP is limited among Asians due to regional cultural influences [2]. There is little understanding of the attitudes and preferences for refusing LST and ANH treatment among healthy Asian individuals. This study aimed to comprehend AD preferences among Taiwan individuals in the urban community who sought consultations for ACP one year after the PRAA passed, clarify the consistency and differences in LST or ANH preferences in different clinical conditions, and explore factors affecting LST and ANH preferences.

## Methods

### Participants and data collection

The study protocol was approved by the institutional review board of Taipei City Hospital (file number: TCHIRB-10808008-E), the main designated institutions responsible for implementing the ACP policy mandated by the Taiwanese government. The study is notable for its comprehensive analysis involving a significant number of participants, marking its pioneering nature in Taiwan. In the inaugural year of the PRAA, a total of 11,317 individuals in Taiwan participated ACP consultation. Taipei City Hospital made a significant contribution with 2,337 participants, representing over a fifth of the national total and achieving the highest signing rate nationally.

Among the seven branches of Taipei City Hospital in the capital, five exceeded 1,300 signatories, establishing it as the most effective and prolific hospital in terms of promoting ACP/ADs in Taiwan. All branches of the Taipei City Hospital initiated ACP consultations, including ACP communication, AD signing, and noting reminders on National Health Insurance ID cards, both in the outpatient and inpatient departments, as well as at-home.

We utilized a survey of legally structured AD documents for data collection.

Data was gathered through the ACP counseling process, collecting personal background details verbally, and obtaining the final signed result of AD. Additionally, data were collected from individuals with legal ability, aged at least 20 years, who participated in ACP consultations at the Taipei City Hospital from January 6, 2019, to January 5, 2020. A total of 2,337 participants engaged in the ACP consultation program, with 2,198 people completing the AD.

### Research materials

The AD and ACP consultation records of the patients were examined. Participants in ACP consultations were presented with the option to accept or decline LST and ANH in scenarios involving terminal illness, permanent vegetative state, irreversible coma, severe dementia, and other proclaimed unbearable and incurable diseases. Following to the consultation, individuals can sign ADs, specifying their preferences for accepting or refusing LST and ANH, tailored to five distinct clinical scenarios. If a declarant meets any of the five clinical conditions after making an AD, the medical institution or physicians may partially or fully terminate, withdraw, or withhold the LST and ANH. The options for LST and ANH preferences includes:

1. No decision has been made.
2. Reluctance to receive LST/ANH.
3. Expectation to receive LST/ANH for a specified duration, with the appointed HCA authorized to remove LST/ANH at any time during that period.
4. HCA authorized to make decisions.
5. Willingness to receive LST/ANH.

The ACP consultation records documented the gender, age, welfare entitlement, disease conditions, family caregiving experience, location of ACP consultation, participation of second-degree relatives, and the intention to participate in ACP. Proposed reasons for participation included:

1. Having a disease.
2. Being unmarried.
3. Desiring a good death with dignity.
4. Hearing from the press reports and propagations.
5. Considering planning for the end of life.
6. Having a member of the family has a disease.
7. Being unwilling to let my family take responsibility for decision-making.
8. Not wanting to be a burden to family.

### Data analysis

We assessed differences in preferences for LST and ANH across the five clinical conditions. Additionally, we explored the consistency of LST and ANH choices in these clinical conditions and investigated the relationship between socio-demographic factors and preferences of LST and ANH. The SPSS 22.0 package (IBM Corp, Armonk, NY) was utilized for data analysis. Descriptive statistics were employed to characterize the nominal and ordinal variables, as well as normally distributed continuous variables. Kappa coefficients were calculated to determine consistency, and various statistical tests, including McNemar-Bowker test, chi-square, Fisher’s exact tests, independent sample t-test, and bivariate and multivariate logistic regressions, were conducted where appropriate. Except for the participants who did not decide and refuse the LST and ANH, we grouped participants who partially or fully received LST and ANH and authorized an HCA for AD as one single group for bivariate and multivariate logistic regressions for LST and ANH preferences.

## Results

### Distribution of LST and ANH preferences

A total of 2,337 participants expressed immediate attitudes during ACP for the five clinical conditions (Table 1). The percentage of participants refusing all treatments ranged from 87.5% to 90.9%. Additionally, 7.2% of the participants had not decided; 4.2% and 3.6% of the participants chose time-limited treatment for terminal diseases and the proclaimed unbearable/incurable diseases, respectively. Regarding ANH preferences, 87.6% to 90.7% of the participants chose to refuse ANH, with 7.2% undecided; 4.0% and 3.5% of the participants accepted time-limited treatment for terminal disease and proclaimed unbearable/incurable diseases. Overall, choices for LST and ANH demonstrated consistent patterns.

**Table 1:** Advance decisions of life-sustaining treatment and artificial nutrition/hydration

	AD five clinical conditions									
	Terminal diseases		Irreversible coma		Sustained vegetative		Severe debilitating dementia		Proclaimed incurable diseases	
	*n*	%	*n*	%	*n*	%	*n*	%	*n*	%
**Preferences of LST**										
Refuse all treatment	2045	87.5%	2096	89.7%	2124	90.9%	2113	90.4%	2051	87.8%
Undecided	168	7.2%	168	7.2%	168	7.2%	168	7.2%	172	7.4%
Authorized HCA to decide	23	1.0%	24	1.0%	20	0.9%	18	0.8%	27	1.2%
Time-limited treatment	98	4.2%	48	2.1%	25	1.1%	35	1.5%	83	3.6%
Accept all treatment	3	0.1%	1	0.0%	0	0.0%	3	0.1%	4	0.2%
**Preferences of ANH**										
Refuse all treatment	2047	87.6%	2096	89.7%	2119	90.7%	2101	89.9%	2051	87.8%
Undecided	167	7.1%	168	7.2%	168	7.2%	168	7.2%	171	7.3%
Authorized HCA to decide	20	0.9%	23	1.0%	19	0.8%	17	0.7%	27	1.2%
Time-limited treatment	93	4.0%	46	2.0%	27	1.2%	43	1.8%	81	3.5%
Accept all treatment	10	0.4%	4	0.2%	4	0.2%	8	0.3%	7	0.3%

### Consistency and differences of LST and ANH preferences among five clinical conditions

Table 2 illustrates that preferences for LST and ANH were consistent across different clinical conditions (Kappa coefficients > 0.783, Kappa coefficients > 0.814). Remarkably, higher consistencies were observed in the clinical conditions of irreversible coma, permanent vegetative state, and severe dementia (Kappa coefficients > 0.9).

**Table 2:** Consistency of the will of LST and ANH at different clinical conditions

**Preferences of LST**	Terminal diseases	Irreversible coma	Sustained vegetative	Severe debilitating dementia	Proclaimed unbearable/incurable
Terminal diseases		0.804	0.783	0.812	0.850
Irreversible coma			0.924	0.902	0.859
Sustained vegetative				0.916	0.817
Severe debilitating dementia					0.849
proclaimed unbearable/incurable diseases					
**Preferences of ANH**	Terminal diseases	Irreversible coma	Sustained vegetative	Severe debilitating dementia	Proclaimed unbearable/incurable
Terminal diseases		0.844	0.814	0.843	0.870
Irreversible coma			0.914	0.900	0.866
Sustained vegetative				0.905	0.825
Severe debilitating dementia					0.869
proclaimed unbearable/incurable diseases					

Regarding clinical conditions, preferences for LST and ANH in the same clinical conditions showed significant consistency (Kappa coefficients 0.917 to 0.972, Table 3). However, preferences of LST and ANH differed significantly between terminal disease (χ^2^ = 12.581, *p* < 0.05) and extremely severe dementia (χ^2^ = 11.4, *p* = 0.05). These differences might be attributed to the preferences for time-limited treatment and continuing to accept treatment during terminal disease conditions and the preferences for time-limited treatment and wishing not to accept any treatment during the extremely severe dementia condition.

**Table 3:** Tests of consistency and difference of different clinical conditions between LST and ANH

Clinical conditions	Consistency test^a^		Difference test^b^	
	Kappa coefficients	*p*-value	Paired chi-square	*p*-value
Terminal diseases (LST vs. ANH)	0.917	< 0.001	12.581	0.022
Irreversible coma (LST vs. ANH)	0.971	< 0.001	4.143	0.529
Sustained vegetative (LST vs. ANH)	0.972	< 0.001	N/A	N/A
Severe debilitating dementia (LST vs. ANH)	0.960	< 0.001	11.400	0.05
proclaimed unbearable/incurable diseases (LST vs. ANH)	0.969	< 0.001	3.077	0.545

^a^denotes Kappa coefficient

^b^denotes McNemar-Bowker test

### Comparisons between the five clinical conditions and factors influencing LST or ANH choices

Table 4 indicates comparisons between the five clinical conditions revealed noteworthy distinctions in time-limited preference for LST and authorized HCA to decide for ANH. Concerning LST preferences, there was a significant difference emerged (χ^2^ = 68.215, *p* < 0.001) in the inclination towards time-limited treatment for terminal diseases (4.2% > 2.1%,4.2% > 1.5%, 4.2% > 1.1%) and proclaimed unbearable/incurable diseases (3.6% > 2.1%,3.6% > 1.5%, 3.6% > 1.1%). Similarly, for ANH preferences, a significant difference was found (χ^2^ = 53.172, *p* < 0.001) in the time-limited treatment related to terminal diseases (4% > 2%,4.2% > 1.8%, 4.2% > 1.2%) and proclaimed unbearable/incurable diseases (3.5 > 2%,3.5% > 1.8%, 3.5% > 1.2%). Additionally, a notable difference in the preference for authorized HCA to decide for ANH (χ^2^ = 21.77, *p* < 0.000) was noted, originated from an irreversible coma and the proclaimed unbearable/incurable diseases. Accordingly, a higher proportion of participants exhibited a tendency to choose time-limited treatment for both LST and ANH when facing terminal diseases and proclaimed unbearable/incurable diseases. Simultaneously, more participants leaned towards allowing the authorized HCA to decide on ANH treatment in the clinical conditions of irreversible coma and unbearable and incurable diseases.

**Table 4:** Distinctions in participants' preferences for LST and ANH among five clinical conditions

Preferences	LST		ANH	
The Wills of LST/ANH	χ2	*p-*value	χ2	*p*-value
Wish not to accept	2.483	0.643	1.973	0.741
Undecided	0.076	0.999	0.055	0.999
Authorized HCA to decide	2.196	0.700	^C^21.77^***^	0.000
Time-limited treatment	^a^68.215^***^	0.000	^b^53.172^***^	0.000
Continue to accept treatment	2.000	0.736	4.121	0.390

^a^and ^b^The significant differences of time-limited treatment preference for both LST and ANH resulted from the clinical conditions of terminal disease and proclaimed unbearable/incurable diseases. ^c^The significant difference of ANH authorized HCA to decide preference was contributed from the clinical conditions of irreversible coma and proclaimed unbearable/incurable diseases

*p* < .05^*^, p < .01^**^, p < .001^***^

### Factors influencing LST or ANH choices: gender, age, and ACP progression

Significant differences were observed in gender, age, the location where ACP progressed, HCA appointment, and intention of ACP, including preferences related to family responsibility (Table 5). Initially, females and older individuals were more inclined to refuse LST and ANH, while those below 40 years old preferred receiving and authorizing the HCA for further decision. Female caregivers demonstrated a higher likelihood of refusing LST and ANH. The proportions of outpatient clinic-based ACP progressions were significantly higher in each LST preference than in other locations. In preferences of refusal, remaining undecided, or receiving LST, more participants were without HCA appointments than those with HCA appointments. Lastly, participants expressing reluctance for family members to take responsibility and desire not to be a family burden exhibited a stronger intention to refuse LST and ANH.

**Table 5:** Factors associated with preference of LST and ANH

Characteristic	Preference of LST								Preference of ANH							
	Refuse LST at five clinical conditions (*n* = 2004, 85.8%)		Undecided instantly (*n* = 166, 7.1%)		Receive LST and authorize HCA (*n* = 167, 7.1%)				Refuse ANH at five clinical conditions (*n* = 2013, 86.1%)		Undecided instantly (*n* = 166, 7.1%)		Receive ANH and authorize HCA (*n* = 158, 6.8%)			
	*n*	%	*n*	%	*n*	%	*p*-value		*n*	%	*n*	%	*n*	%	*p*-value	
Gender							** < 0.001**	^a^ ***							** < 0.001**	^a^ ***
Male	664	33.1%	65	39.2%	83	49.7%			665	33.0%	65	39.2%	82	51.9%		
Female	1340	66.9%	101	60.8%	84	50.3%			1348	67.0%	101	60.8%	76	48.1%		
Age																
Below 40 years	191	9.5%	20	12.0%	31	18.6%	**0.003**	^a^ **	192	9.5%	20	12.0%	30	19.0%	**0.003**	^a^ **
41–65	958	47.8%	84	50.6%	79	47.3%			964	47.9%	84	50.6%	73	46.2%		
Above 65 years	855	42.7%	62	37.3%	57	34.1%			857	42.6%	62	37.3%	55	34.8%		
Family caregiving experience							0.053	^a^							**0.044**	^a^ *
No	900	59.9%	54	52.4%	80	68.4%			904	59.9%	54	52.4%	76	69.1%		
Yes	602	40.1%	49	47.6%	37	31.6%			605	40.1%	49	47.6%	34	30.9%		
**Caregiver's gender**							**0.036**	^a^ *							**0.036**	^a^ *
Male	164	27.2%	20	40.8%	15	40.5%			165	27.3%	20	40.8%	14	41.2%		
Female	438	72.8%	29	59.2%	22	59.5%			440	72.7%	29	59.2%	20	58.8%		
**Welfare entitlement**							0.438	^a^							0.235	^a^
General public (no welfare entitlement)	1760	87.8%	151	91.0%	145	86.8%			1771	88.0%	151	91.0%	134	84.8%		
With welfare entitlement (all)	244	12.2%	15	9.0%	22	13.2%			242	12.0%	15	9.0%	24	15.2%		
**Disease severity**							0.204	^a^							0.079	^a^
No self-reported diseases	1082	79.7%	73	73.0%	92	76.0%			1092	79.9%	73	73.0%	82	73.2%		
With diseases (clinical conditions related (all)	276	20.3%	27	27.0%	29	24.0%			275	20.1%	27	27.0%	30	26.8%		
**Disease conditions- types of disease**																
Cancers	113	9.5%	8	9.9%	8	8.0%	0.880	^a^	114	9.5%	8	9.9%	7	7.9%	0.873	^a^
stroke history or cardiovascular diseases	63	5.5%	8	9.9%	3	3.2%	0.143	^a^	63	5.5%	8	9.9%	3	3.5%	0.191	^b^
Mental diseases	57	5.0%	4	5.2%	8	8.0%	0.436	^a^	55	4.8%	4	5.2%	10	10.9%	0.058	^b^
Neurodegenerative diseases	39	3.5%	4	5.2%	6	6.1%	0.336	^a^	38	3.4%	4	5.2%	7	7.9%	0.068	^b^
Liver cirrhosis and any organ failure	36	3.2%	5	6.4%	4	4.2%	0.309	^a^	37	3.3%	5	6.4%	3	3.5%	0.294	^b^
**The place ACP progressed**							**0.034**	^b^ *							**0.010**	^b^ *
Hospital (outpatient clinic)	1918	95.7%	161	97.0%	154	92.2%			1929	95.8%	161	97.0%	143	90.5%		
Hospital (admission)	40	2.0%	0	0.0%	9	5.4%			40	2.0%	0	0.0%	9	5.7%		
Home	18	0.9%	2	1.2%	1	0.6%			17	0.8%	2	1.2%	2	1.3%		
Institution	28	1.4%	3	1.8%	3	1.8%			27	1.3%	3	1.8%	4	2.5%		
**Participation of second-degree relatives**							0.199	^a^							0.260	^a^
No	215	12.0%	18	14.6%	11	7.7%			215	11.9%	18	14.6%	11	8.1%		
Yes	1582	88.0%	105	85.4%	131	92.3%			1589	88.1%	105	85.4%	124	91.9%		
**HCA appointment**							** < 0.001**	^a^ ***							** < 0.001**	^a^ ***
No	1809	90.6%	127	81.9%	116	70.3%			1819	90.6%	127	81.9%	106	68.4%		
Yes	188	9.4%	28	18.1%	49	29.7%			188	9.4%	28	18.1%	49	31.6%		
**Intention of ACP**																
Own disease suffering	140	7.0%	13	7.9%	16	9.6%	0.431	^a^	139	6.9%	13	7.9%	17	10.8%	0.182	^a^
Being single	199	10.0%	13	7.9%	13	7.8%	0.488	^a^	203	10.1%	13	7.9%	9	5.7%	0.147	^a^
Expecting a good end with dignity	1342	67.1%	104	63.0%	102	61.4%	0.207	^a^	1343	66.9%	104	63.0%	101	64.3%	0.509	^a^
Prior life arrangement	1274	63.7%	95	57.6%	107	64.5%	0.275	^a^	1280	63.7%	95	57.6%	101	64.3%	0.277	^a^
Media reports and propagations	130	6.5%	10	6.1%	12	7.2%	0.907	^a^	130	6.5%	10	6.1%	12	7.6%	0.823	^a^
Suffering of family members	261	13.1%	28	17.0%	30	18.1%	0.087	^a^	266	13.2%	28	17.0%	25	15.9%	0.287	^a^
Do not wish family members to take responsibility for making decisions	912	45.6%	74	44.8%	55	33.1%	**0.008**	^a^ **	917	45.7%	74	44.8%	50	31.8%	**0.004**	^a^ **
Do not want to be a family drag	815	40.8%	55	33.3%	51	30.7%	**0.009**	^a^ **	818	40.7%	55	33.3%	48	30.6%	**0.010**	^a^ *

^a^Chi-square test

^b^Fisher exact test

^*^p < 0.05

^**^p < 0.01

^***^p < 0.001

### Correlation of gender, age, and consultation intention with refusal of LST and ANH

Significant differences in gender, age, and consultation intention of not wanting family members to take responsibility were observed (Table 6). For LST preferences, female (AOR = 1.679, *p* < 0.05), individuals aged 41 to 64 years (AOR = 2.205, *p* < 0.01), those aged above 65 years (AOR = 2.630, *p* < 0.01), and those with the consultation intention of not wishing family members to take responsibility (AOR = 2.112, *p* < 0.01) were significantly correlated with refusing LST. Similarly, for ANH preferences, females (AOR = 1.673, *p* < 0.05), individuals aged above 65 years (AOR = 2.561, *p* < 0.01), and those with the consultation intention of not wishing family to take responsibility (AOR = 1.721, *p* < 0.05) were significantly correlated with refusing ANH.

**Table 6:** Multivariate logistic regression—factors associated with preference of LST and ANH

Preference of LST	Reference group: Receive LST and authorize HCA								Reference group: Refuse LST at five clinical conditions							
	Model 1. Refuse LST (*n* = 2004)				Model 2. Undecided instantly (*n* = 166)				Model 3. Undecided instantly (*n* = 166)				Model 4. Receive LST and authorize HCA (*n* = 167)			
	Adjusted-OR	95%CI		*p*-value	Adjusted-OR	95%CI		*p*-value	Adjusted-OR	95%CI		*p*-value	Adjusted-OR	95%CI		*p*-value
Gender (ref.: male)																
Female	1.679	1.128	2.500	0.011	1.103	0.636	1.913	0.728	0.657	0.434	0.993	0.046	0.595	0.400	0.886	0.011
Age (ref.: below 40 years)																
41–65 years	2.205	1.282	3.793	0.004	2.621	1.093	6.281	0.031	1.188	0.569	2.483	0.646	0.454	0.264	0.780	0.004
Above 65 years	2.630	1.503	4.603	0.001	2.365	0.959	5.833	0.062	0.899	0.421	1.922	0.784	0.380	0.217	0.665	0.001
Caregiving experience (ref.: No)																
Yes	1.421	0.906	2.229	0.126	1.983	1.091	3.606	0.025	1.396	0.911	2.140	0.126	0.704	0.449	1.104	0.126
The place ACP progressed (ref.: outpatient clinic)																
Hospital admission/home/institution	0.580	0.277	1.214	0.149	0.463	0.136	1.575	0.218	0.797	0.282	2.256	0.669	1.723	0.824	3.605	0.149
HCA appointment (Ref.: No)																
Yes	0.216	0.135	0.347	< 0.001	0.368	0.178	0.761	0.007	1.699	0.915	3.157	0.093	4.620	2.884	7.399	< 0.001
Intention of consultation—own disease suffering (ref.: No)																
Yes	0.789	0.414	1.505	0.472	0.844	0.327	2.181	0.727	1.070	0.502	2.279	0.861	1.267	0.664	2.418	0.472
Intention of consultation—disease suffering of family members (ref.: No)																
Yes	0.685	0.398	1.179	0.172	0.679	0.319	1.444	0.315	0.991	0.560	1.752	0.974	1.459	0.848	2.510	0.172
Intention of consultation—wish not family members to take responsibility for making decision (ref.: No)																
Yes	2.112	1.382	3.228	0.001	1.761	0.997	3.111	0.051	0.834	0.554	1.256	0.384	0.473	0.310	0.724	0.001
Preference of ANH	Reference group: Receive ANH and authorize HCA								Reference groups: Refuse ANH preference at five clinical conditions							
	Model 5. Refuse ANH at five clinical conditions (*n* = 2013)				Model 6. Undecided instantly (*n* = 166)				Model 7. Undecided instantly (*n* = 166)				Model 8. Receive ANH and authorize HCA(*n* = 158)			
	Adjusted-OR	95%CI		*p*-value	Adjusted-OR	95%CI		*p*-value	Adjusted-OR	95%CI		*p*-value	Adjusted-OR	95%CI		*p*-value
Gender (ref.: male)																
Female	1.673	1.076	2.603	0.022	1.607	1.018	2.537	0.042	0.961	0.523	1.764	0.897	0.598	0.384	0.930	0.022
Age (ref.: below 40 years)																
41-65 years	1.275	0.775	2.100	0.339	1.263	0.774	2.060	0.349	0.990	0.506	1.939	0.978	0.784	0.476	1.291	0.339
Above 65 years	2.561	1.350	4.857	0.004	0.884	0.370	2.110	0.781	0.345	0.122	0.974	0.045	0.391	0.206	0.741	0.004
Caregiving experience (ref.: no)																
Yes	1.266	0.774	2.071	0.348	0.811	0.505	1.302	0.386	0.641	0.332	1.235	0.184	0.790	0.483	1.292	0.348
Welfare entitlement (ref.: general public)																
Yes	0.970	0.467	2.016	0.936	0.361	0.138	0.944	0.038	0.372	0.116	1.193	0.096	1.031	0.496	2.141	0.936
Disease condition (ref.: no self-reported diseases)																
With diseases (clinical conditions related—all)	1.317	0.656	2.642	0.439	2.074	1.061	4.054	0.033	1.575	0.626	3.963	0.334	0.760	0.379	1.524	0.439
The place ACP progressed (ref.: hospital outpatient clinic)																
Hospital admission/home/institution	0.464	0.203	1.060	0.068	3.075	0.407	23.212	0.276	6.630	0.780	56.358	0.083	2.156	0.944	4.927	0.068
HCA appointment (ref.: no)																
Yes	0.172	0.103	0.286	< 0.001	0.443	0.233	0.843	0.013	2.580	1.208	5.509	0.014	5.824	3.494	9.709	< 0.001
Intention of consultation—own disease suffering (ref.: no)																
Yes	0.792	0.353	1.780	0.573	1.442	0.498	4.174	0.500	1.819	0.507	6.532	0.359	1.262	0.562	2.834	0.573
Intention of consultation—disease suffering of family members (ref.: no)																
Yes	0.962	0.497	1.861	0.908	1.017	0.529	1.953	0.960	1.057	0.435	2.572	0.902	1.040	0.537	2.012	0.908
Intention of consultation—do not wish family members to take responsibility for making decisions (ref.: no)																
Yes	1.721	1.087	2.725	0.021	1.041	0.662	1.634	0.863	0.605	0.326	1.123	0.111	0.581	0.367	0.920	0.021

## Discussion

This study unveiled a consistent trend in willingness expressions across five hypothetical clinical conditions, with over 90% of participants choosing to decline both LST and ANH. The highest refusal percentage was observed in the permanent vegetative state, demonstrating a pronounced inclination against interventions in scenarios characterized by severe cognitive impairment. This reluctance to accept LST and ANH persisted notably in the permanent vegetative state, severe dementia, and irreversible coma. More participants expressed a desire to decline treatment in the cases involving the permanent vegetative state, severe dementia, and irreversible coma, compared to scenarios with terminal diseases. Notably, in cases of terminal disease, a higher proportion of participants favored time-limited treatment for both LST and ANH.

The consideration of rejecting LST treatment, primarily in the context of terminal diseases, has not extended to conditions such as the permanent vegetative state, severe dementia, and irreversible coma [4]. Unlike patients facing terminal diseases who typically retain mental capacity, those in a permanent vegetative state, severe dementia, or enduring irreversible coma, lack the autonomy to make decisions independently. Consequently, some countries have embraced proactive approaches to make medical decisions in advance, aiming to enhance the prevalence of autonomous decisions [4]. Notably, in Taiwan, neurological diseases like the permanent vegetative state, severe dementia, and irreversible coma, initially not considered terminal among Asians [10], have gradually been added to the list of terminating illnesses. These newly incorporated diseases in Taiwan’s PRAA relate to neurological diseases that are highly likely to induce incapacity and dependence, causing cognitive impairment, reliance on others for care, and a diminished quality of life [11].

A nationwide population-based study in Taiwan highlighted that healthcare burden associated with dementia, revealing higher rates of hospitalization, intensive care unit admissions, and extended stays than cancer patients [10]. Except for blood transfusions, the prevalence of LST and ANH use was significantly greater in dementia patients than in cancer patients. Additionally, the utilization of ANH exceeded that of LST, including the additional requirements such as enteral tube insertion (72.6%), feeding (67.4%), mechanical ventilation (61.5%), endotracheal intubation (59.6%), cardiopulmonary resuscitation (33.9%), and hemodialysis (17.6%) [10]. Furthermore, the prevalence of tube feeding or enteral tube insertion in the dementia patients in Taiwan was significantly higher than in Europe (20.5% in Italy), North America (25% in the USA and 11% in Canada), and other Asian regions (66% in Hong Kong) [10].

In comparison to LST, participants showed a higher acceptance of ANH as a time-limited treatment, along with a preference for authorizing an HCA for subsequent decisions. In the case of irreversible coma, a higher number of participants inclined toward preferring an authorized HCA to make decisions about ANH. In conditions of severe dementia and terminal diseases, more participants were open to accepting time-limited ANH treatment. Regarding proclaimed unbearable/incurable disease, more participants authorized the HCA to decide on ANH.

The preference of LST and ANH can be influenced by various factors, including culture, religion, tradition, value and beliefs, administrative guidelines, and the dynamics of the doctor– family–patient relationships [1, 12–14]. Some studies have highlighted the challenges in providing ANH to the end-of-life patients [15, 16]. Patients may require artificial nutrition for a variety of reasons, such as survival, feeling better, or maintaining appearances for the sake of their family [3, 17]. For instance, artificial nutrition serves as essential support for comatose patients and those in a persistent vegetative state, bridging the gap until recovery becomes either imminent or unlikely [16]. Late-stage dementia is characterized by a loss of ability and desire to eat, causing emotional distress for relatives when patients reduce oral intake [16, 17]. Conflicting perspectives exist regarding ANH, viewing it either as a fundamental aspect of basic nursing care or as a medical therapy that still lacks clear indications [3, 18].

This study revealed that the social-demographic characteristics of the participants had significantly influenced their preferences for LST and ANH. Generally, females tended to outright refuse both LST and ANH, without expressing indecision, and they did not opt for time-limited treatment, authorizing the HCA, or receiving treatments. In contrast, males tended to receive the full or time-limit treatment. The gender difference in LST and ANH preferences observed in our study is aligns with previous studies on gender difference in palliative care preferences and treatments [19–22]. The societal perception that diseases as wars, with treatments symbolizing battles and aspirations for cures framed as fights, might motivate men to confront and combat these diseases [19, 23]. On the other hand, the social values afford women more space for sentimentality, expressing symptoms, and seek social assistance [19, 24].

Additionally, the study’s findings indicate that participants currently signing AD typically did not have significant illnesses, as over 73% reported no self-reported diseases or non-life-threatening chronic diseases. The decision to sign AD was based on their contemplation of five hypothetical clinical scenarios. There was a significant correlation between the decisions to sign AD and participants’ age, suggesting that age influences their contemplation, attitudes, and decisions. Participants under the age of 40 tended to opt for receiving full or time-limit treatment and authorizing an HCA for subsequent decisions rather than refusing outright. Those between the ages of 40 and 65 often remained undecided, while participants over 65 tended to refuse the full or time-limit treatment. This age-related trend aligns with findings indicating a positive association between age and AD signing in nursing homes and cancer patients [25], with older patients more commonly having DNAR orders [26].

Furthermore, two significant family-related factors contributing to the refusal of LST and ANH treatment were the reluctance of family members to assume responsibility and the rejection of HCA appointment. This mirrors the prevalent ACP issues in Asian culture, which primarily revolve around family-related concerns [6, 15]. Sun et al. reported instances in which ICU surrogates faced emotional interference from families with conflicting views on medical treatment, thereby influencing decision-making [27]. With the implementation of PRAA, we anticipate a better understanding of and emphasis on patient autonomy, enabling physicians to provide more accurate diagnoses and engage in more direct communication with patients.

### Research limitations

The study exclusively investigated immediate preferences concerning ADs during ACP consultations. The research scope did not extend to subsequent alterations in choices or discussions post-consultations. Furthermore, participants were selected from Taipei City Hospital, designated as the primary trial and demonstration site for ACP in Taipei City. The exclusive focus on patients from one hospital imposes constraints on the external validity of the findings.

### Implication

The findings provide insights into tailoring ACP consultation methods for ANH, considering social and cultural nuances. Adaptable and sensitive approaches can address diverse public needs, including those resistant to ACP consultations. Future research avenues may explore how medical choices evolve with changing health statuses and identify determinants influencing the duration of time-limiting treatments. Further investigation into the perspectives and attitudes of Taiwanese medical personnel regarding the removal of LST and ANH for patients with neurological diseases could enhance our understanding.

## Conclusion

The study examined urban residents’ preferences for LST or ANH across different clinical conditions. Consistent patterns emerged in preferences for LST and ANH, particularly in irreversible coma, permanent vegetative state, and severe dementia. However, differences were observed in terminal disease and extremely severe dementia. Preferences for time-limited treatment and HCA decision-making varied across conditions, with more participants opting for time-limited treatment in terminal and proclaimed unbearable/incurable diseases. Gender, age, and ACP progression significantly influenced preferences, with females and older individuals more likely to refuse treatment. Younger participants preferred authorizing the HCA for decision-making. Additionally, factors such as outpatient clinic-based ACP progressions, HCA appointments, and intentions related to family responsibility were associated with participants’ preferences for LST and ANH. Overall, the study underscores the importance of considering individual preferences and factors in advance care planning discussions, especially regarding LST and ANH preferences among urban residents with various clinical conditions. Tailored approaches are essential for effective end-of-life care decision-making.

## Data Availability

The data that support the findings of this study are available from the Department of Social Work, Taipei City Hospital, but restrictions apply to the availability of these data, which were used under license for the current study, and so are not publicly available. Data are however available from the authors upon reasonable request and with permission of the Department of Social Work, Taipei City Hospital.

## Abbreviations

ACP: Advance care planning
AD: Advance decision
ANH: Artificial nutrition and hydration
EoL: End of life
HCA: Health care agent
HPCA: Hospice Palliative Care Act
LST: Life-sustaining treatment
MHW: Ministry of Health and Welfare
PRAA: Patient Right to Autonomy Act
